# MWCNT-Supported PVP-Capped Pd Nanoparticles as Efficient Catalysts for the Dehydrogenation of Formic Acid

**DOI:** 10.3389/fchem.2020.00359

**Published:** 2020-04-28

**Authors:** Alejandro Ortega-Murcia, Miriam Navlani-García, Emilia Morallón, Diego Cazorla-Amorós

**Affiliations:** ^1^Physical Chemistry Department, Institute of Materials Science (IUMA), University of Alicante (UA), Alicante, Spain; ^2^Inorganic Chemistry Department, Institute of Materials Science (IUMA), University of Alicante (UA), Alicante, Spain

**Keywords:** hydrogen, formic acid, palladium nanoparticles, MWCNT, polyvinylpyrrolidone, capping agent

## Abstract

Various carbon materials were used as support of polyvinylpyrrolidone (PVP)-capped Pd nanoparticles for the synthesis of catalysts for the production of hydrogen from formic acid dehydrogenation reaction. Among investigated, MWCNT-supported catalysts were the most promising, with a TOF of 1430 h^−1^ at 80°C. The presence of PVP was shown to play a positive role by increasing the hydrophilicity of the materials and enhancing the interface contact between the reactant molecules and the catalytic active sites.

## Introduction

Formic acid, the simplest carboxylic acid (HCOOH, FA), has recently received great interest as a hydrogen carrier molecule (Enthaler et al., 2010; Grasemann and Laurenczy, 2012; Navlani-García et al., 2019a; Valentini et al., 2019). The increasing literature reporting on that topic is particularly notorious in the last decade, when numerous approaches to design efficient catalysts to boost the dehydrogenation of FA have been considered. That reaction produces H_2_ and CO_2_ in a molar ratio of 1 to 1. However, the side dehydration reaction is responsible for the formation of CO, and hence should be avoided, which motivated the search for selective catalysts. Among the possible compositions of the catalysts, Pd-based heterogeneous catalysts have been the most investigated so far, which is due to their suitable performance under mild conditions, in terms of both conversion and selectivity. Many aspects have already been tackled, which encompass the optimization of the morphology of the nanoparticles (Navlani-García et al., 2015b, 2016b) and their composition (Mori et al., 2013; Qin et al., 2013; Wang et al., 2013; Navlani-García et al., 2016c, 2018a), as well as the evaluation of the properties of the support material (Navlani-García et al., 2015a; Mori et al., 2017a; Wu et al., 2017). At this point, it is already well-known and widely reported that catalysts with basic character tend to result in enhanced performance toward the dehydrogenation of FA, which is either ascribed to their interaction with FA molecules or the stabilization of metal nanoparticles with small size and narrow size distributions (Mori et al., 2013; Navlani-García et al., 2018b). The incorporation of nitrogen functional groups has widely been reported as a fruitful strategy to achieve high-performance catalysts and it has been applied to supports of diverse nature [i.e., carbon materials (Bulushev et al., 2016a,b; Navlani-García et al., 2018b; Podyacheva et al., 2018; Golub et al., 2019; Sun et al., 2019), silica (Mori et al., 2019), resins (Mori et al., 2015, 2017b), metal-organic frameworks (Wen et al., 2017), etc.]. However, due to their versatility and outstanding features, carbon materials have been the most extensively investigated supports so far.

Concerning the synthetic approaches used for the preparation of N-doped carbon materials, they can be classified as *in-situ* and post-synthesis doping methods, depending on whether the N-doping and the synthesis of the carbon material take place in one or in consecutive steps (Salinas-Torres et al., 2019). The preparation of N-doped carbon material-supported metal catalysts frequently requires multiple synthesis steps (i.e., preparation of the carbon material, incorporation of N-functionalities, metal loading, metal reduction, etc.).

The standard impregnation method commonly used in the synthesis of metal nanoparticles frequently results in large nanoparticles and/or wide nanoparticles size distribution, which hampers the proper assessment of the catalytic activity. In this line, the synthesis of colloidal nanoparticles has been postulated as a useful tool for the preparation of size and shape-controlled nanoparticles with narrow size distribution (Navlani-García et al., 2019b). In particular, the reduction-by-solvent method [so-called polyol method (Fiévet et al., 2018)] has fruitfully been applied for the preparation of noble metal nanoparticles used in countless applications (i.e., hydrogenation reactions Domínguez-Domínguez et al., 2006, 2008; Tsung et al., 2009; Navlani-García et al., 2017, preferential CO oxidation reaction Miguel-García et al., 2015; Navlani-García et al., 2016a, biosensing Quintero-Jaime et al., 2019, carbon-carbon bond-forming reactions Ohtaka et al., 2015, etc.). Such a method usually requires the use of a capping agent, mainly polymers, which serve as a stabilizing agent. Moreover, the capping agent can also modify the properties of the resulting metal nanoparticles due to the interaction with the different functional groups present in the polymer molecules. Among all the possible capping agent, poly(N-vinyl-2-pyrrolidone) (PVP) is the most extensively used in the synthesis of colloidal metal nanoparticles, which is due to its higher protective character as compared to that of other polymers (Navlani-García et al., 2019b). Due to the chemical composition of its monomers (N-vinylpyrrolidone), PVP can strongly interact with the resulting capped nanoparticles.

The present study tackles the development of nitrogen-containing Pd-based carbon-supported catalysts by a simple and scalable synthetic protocol. Here, we address for the first time the use of PVP-capped Pd nanoparticles utilized as a strategy to synthesize N-containing Pd-based catalysts for the dehydrogenation of FA. Furthermore, the effect of the carbon material was also checked by using alternative supports to the most commonly investigated activated carbon [i.e., carbon black and multi-walled carbon nanotubes (MWCNTs)]. The counterpart PVP-free catalysts were prepared to check the effect of N-containing polymer in the system. The present study might pave the way for the future development of new catalysts in which the surfactants not only act as stabilizing agents in the synthesis of the metal nanoparticles, but they can also participate in the incorporation of new functional groups to the resulting catalysts.

## Experimental

### Synthesis of PVP-Capped Pd Nanoparticles

The synthesis of PVP-capped Pd nanoparticles was performed by a well-reported methodology (Domínguez-Domínguez et al., 2006; Miguel-García et al., 2010), using Pd(OAc)_2_ as the metal precursor, polyvinylpyrrolidone (PVP, 40 K) as a capping agent, and ethylene glycol as both solvent and reducing agent. The PVP/Pd molar ratio used in the synthesis was 10/1 and the reduction temperature was fixed at 100°C.

### Preparation of Carbon-Supported Catalysts

Supported catalysts were prepared by the impregnation method with the as-synthesized nanoparticles and the selected supports (CD-6008 carbon black (CB) from COLUMBIAN CHEMICALS, commercial XC-72F Vulcan carbon black (Vulcan) from Cabot Corporation, and multiwall carbon nanotubes (MWCNT) from COLUMBIAN CHEMICALS). For that purpose, the supports were added to the adequate volume of colloidal nanoparticles to have a final metal loading of 3 wt.% and the mixtures were stirred for 2 days at room temperature. After that, the solvent was evaporated at 60°C and the collected solids were washed several times with a mixture of ethanol/water (50/50% v/v). The samples were dried overnight, and the resulting catalysts based on CB, Vulcan, and MWCNT were denoted as Pd/CB, Pd/Vulcan, and Pd/MWCNT, respectively. To assess the effect of PVP molecules on the final catalytic performance, the counterpart PVP-free catalysts were prepared. For that, Pd/CB, Pd/Vulcan, and Pd/MWCNT catalysts were treated at 450°C (with a heating rate of 10°C/min) under nitrogen atmosphere for 10 min. The obtained PVP-free catalysts were denoted as Pd/CB(t), Pd/Vulcan(t), and Pd/MWCNT(t), respectively.

### Characterization

The textural characterization of the samples was carried out using N_2_ adsorption at −196°C (Autosorb 6, Quantachrome). Before the adsorption measurements, the samples were outgassed under vacuum at 200°C for 4 h to remove any possible adsorbed impurity. Apparent surface area values and total micropore volumes (V_DR_) were calculated using BET equation (S_BET_) and the Dubinin–Radushkevich (DR) equation, respectively. Thermogravimetric analysis was performed using a TA Instruments SDT Q600 thermobalance and with a N_2_ flow of 100 mL/min and heating the catalysts up to 450°C and keeping that temperature for 20 min. Transmission electron microscopy (TEM) images of the catalysts were obtained by using a JEOL (JEM-2010) transmission electron microscope equipped with an EDS analyzer (OXFORD, model INCA Energy TEM 100) operating at 200 kV with a space resolution of 0.24 nm. Palladium content was determined by ICP-OES (inductively coupled plasma-optical emission spectroscopy) with a Perkin-Elmer Optima 4,300 system. The metal extraction was performed by oxidative treatment with aqua regia for 48 h. XPS (X-ray photoelectron spectroscopy) analysis was carried out in a VG-Microtech Multilab 3000 spectrometer equipped with a semispherical electron analyzer and a Mg Kα (hν = 1253.6 eV) 300 W X-ray source. Binding energies were referenced to the C 1s line at 284.6 eV. Pd(0) and Pd(II) relative contents were determined from the integrated intensities of the spectra.

### Catalytic Test

The catalytic activity toward the decomposition of FA was evaluated by monitoring the gas evolution profiles achieved with the time while performing the reaction at 80°C. For that, 0.15 g of powder catalyst were placed in a reactor and a solution of formic acid and sodium formate, in a molar ratio of 9/1 and total concentration of 1 M, was added. The gas produced was measured by using a burette system. TOF values (h^−1^) were calculated with the following equation:

(1) $$\begin{array}{l} TOF\;\left(h^{-1}\right)=\frac{produced\;H_{2}\;\left(mole\right)}{Pd\;content\;\left(mole\right)\times time\;\left(h\right)} \end{array}$$

where the H_2_ produced (mole) was measured after 1 min of reaction and the Pd content (in mole) is the total number of moles added, which was calculated considering the mass of catalyst used in the test and the metal loading determined by ICP-OES analysis. TOF was also calculated considering the mole of surface Pd as determined from TEM. To this end, first, the average Pd nanoparticle diameters (d_TEM_) were calculated for each catalyst by counting a large number of nanoparticles in the TEM micrographs and using the following equation:

(2) $$\begin{array}{l} d_{TEM}\;=\frac{\sum n_{i}d_{i}}{\sum n_{i}} \end{array}$$

From the d_TEM_, Pd nanoparticle dispersion (D_TEM_, defined as the number of metal atoms on the sample surface divided by the total number of metal atoms) was estimated by assuming spherical nanoparticle geometry and using the following equation (Domínguez-Domínguez et al., 2008):

(3) $$\begin{array}{l} D_{TEM}=10^{21}\frac{6{M\rho }_{site}}{\rho_{Pd}Nd_{TEM}} \end{array}$$

Where M is the atomic weight, ρ_site_ is the Pd surface site density, ρ_Pd_ is the metal density, N is the Avogadro constant and d_TEM_ is the average diameter of the nanoparticles.

After that, the value of surface Pd atoms was calculated for each sample by using the following expression, where the real Pd loading determined by ICP was used:

(4) $$\begin{array}{l} Surface\;Pd\;atoms=\frac{{{mole}_{Pd}}^{*}D_{TEM}}{100} \end{array}$$

Finally, TOF values calculated on the bases of Pd surface atoms were calculated as follows:

(5) $$\begin{array}{l} TOF\;\left(h^{-1}\right)=\frac{produced\;H_{2}\;\left(mole\right)}{Surface\;Pd\;atoms\;\left(mole\right)\times time\;\left(h\right)} \end{array}$$

## Results

### Characterization

**Figure 1:**
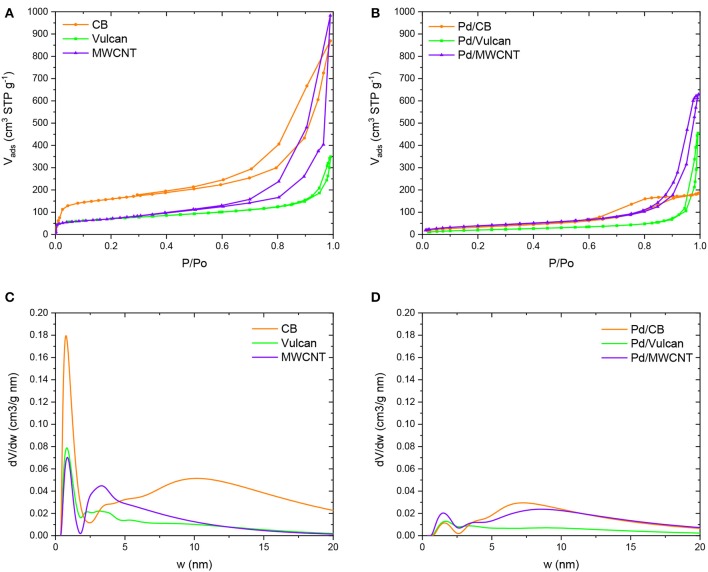
N_2_ adsorption-desorption isotherms of supports **(A)** and catalysts **(B)**; Pore size distribution of supports **(C)** and catalysts **(D)**.

The results of the N_2_ adsorption-desorption isotherms and pore size distributions calculated by NLDFT method considering slit shaped pores, for the three supports and the counterpart catalysts are plotted in Figure 1. Table 1 contains the porous texture parameters determined from the isotherms. As can be seen in Figure 1A, Vulcan shows a Type II isotherm, with a low volume of micropores, while CB and MWCNT have a certain degree of mesoporosity. Interestingly, sample CB has a significant volume of micropores (Table 1). In the case of Pd-containing catalysts (Figure 1B), the isotherms evidenced a significant decrease of porosity upon Pd loading, which might be due to a partial blockage or filling of the pores of the carbon materials due to the presence of Pd nanoparticles. Such observation is in good agreement with the results of the porous texture characterization included in Table 1. As can be seen, S_BET_ values of the Pd-containing catalysts are significantly lower than those of the counterpart supports (S_BET_ decreases around 79, 68, and 42% for CB, Vulcan, and MWCNT-based catalysts, respectively). That effect is also observed in the volumes of micropores and mesopores. Figures 1C,D contain the pore size distribution of supports and Pd-containing catalysts, respectively. In the case of the supports, a bimodal distribution with a large contribution of both micropores (< 2 nm) and mesopores (2–50 nm) was observed. However, as can be seen in the results of V_DR_ and in Figure 1D, the contribution of the micropores considerably decreased after Pd loading and, even though such decrease was also observed for the mesopores, the effect is less marked. This decrease in microporosity is clearly observed for sample CB, whose micropore volume decreases from 0.38 to 0.05 cm^3^/g. Such porosity decrease observed in the catalysts as compared to the counterpart supports might be ascribed to the blockage or filling by Pd nanoparticles, being the blockage the most important contribution in the case of microporosity. It must be taken into account that the Pd nanoparticles are PVP protected and PVP molecules may strongly interact with the supports, especially in the microporosity, and may be detached from the surface of the nanoparticles.

**Table 1:** Results of the characterization of the textural properties.

**Sample**	**S_BET_ (m^2^ g^−1^)**	**V_DR_ (cm^3^ g^−1^)**	**V_meso_ (cm^3^ g^−1^)**
CB	604	0.38	0.42
Pd/CB	126	0.05	0.20
Vulcan	255	0.10	0.12
Pd/Vulcan	79	0.03	0.07
MWCNT	253	0.10	0.29
Pd/MWCNT	146	0.06	0.24

**Figure 2:**
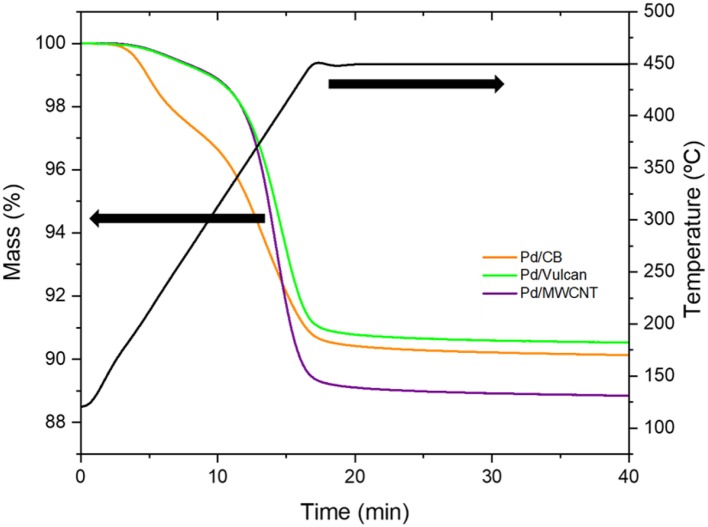
Thermogravimetric analysis profiles performed in N_2_ atmosphere.

Concerning the heat-treated catalysts, the removal of PVP molecules by the heat treatment performed at 450°C was evidenced using thermogravimetric analysis. As observed in the mass loss profiles of the three catalysts under study (Figure 2), a marked mass loss of ~10 wt. % took place upon heating at 450°C, which corresponds to the elimination of PVP molecules from the samples. The results show that the PVP content is similar for the three catalysts (the weight loss after heating at 450°C is between 9.5 and 11 wt. %), what is in agreement with the preparation method in which the same PVP/Pd ratio was used and with the similar Pd content in the catalysts.

**Figure 3:**
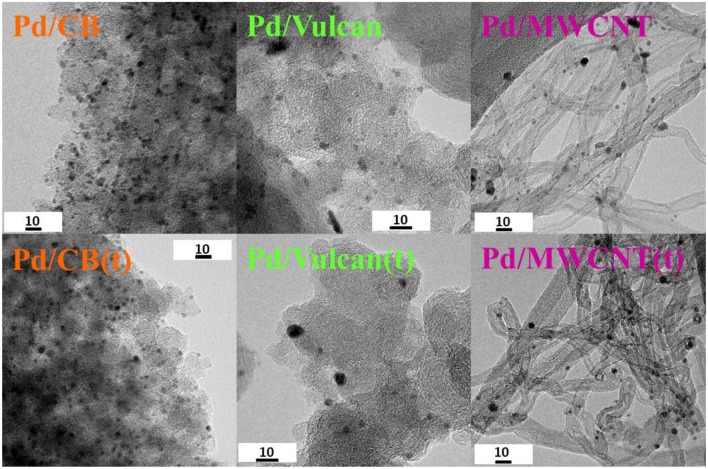
TEM micrographs of the as-synthesized and treated catalysts.

TEM analysis was used to get information about the Pd nanoparticle size and distribution on the different catalysts under study. For that, both sets of as-synthesized and treated catalysts were analyzed to check the possible effect of the heat treatment in the final morphology of the nanoparticles. Figure 3 includes representative micrographs of the two series of catalysts. The average nanoparticle size was determined by counting ~100 nanoparticles for each sample. The colloidal Pd nanoparticles used for the preparation of the catalysts had an average particle size of 2.5 ± 0.7 nm and a very narrow size distribution.

The average nanoparticle size of the studied catalysts is listed in Table 2. The resulting catalysts had an average nanoparticle size of 3.3, 2.6, and 2.5 nm, for Pd/CB, Pd/Vulcan, and Pd/MWCNT, respectively. The nanoparticles are well-dispersed on the three supports (see Figure 3 and Figure S1) and, as was previously reported for Pd-catalysts prepared from colloidal nanoparticles, no aggregation was detected for the supported particles, but a slight increase of the average particle size was observed in some cases along with a partial loss of the spherical shape of the nanoparticles as compared with the unsupported colloidal counterpart. Such observation has already been reported and it is ascribed to the existing interaction between support and nanoparticles (Domínguez-Domínguez et al., 2008; Miguel-García et al., 2010; Navlani-García et al., 2016a). In the case of the treated catalysts, a small increase in the average nanoparticle size took place for Pd/Vulcan(t) and Pd/MWCNT(t), while it remained unchanged for Pd/CB(t) [average nanoparticle size of 3.3, 3.5, and 4.0 nm for Pd/CB(t), Pd/Vulcan(t), and Pd/MWCNT(t), respectively]. That could be related to the larger surface area and micropore volume of CB as compared to the other supports and the subsequent better anchoring of the nanoparticles on its surface. The average Pd nanoparticle size determined in all cases confirmed that, as claimed from the analysis of the porous texture of the materials, some nanoparticles might be located at the entrance of the porosity or even within the porosity of the support, which would ultimately give rise to the observed decreased S_BET_ and pore volumes. As for the Pd loading, 2.7, 3.0, and 2.4 wt. % was determined for Pd/CB, Pd/Vulcan, and Pd/MWCNT, which is close to the nominal metal content (see Table 2).

**Table 2:** Characterization of the catalysts.

**Catalyst**	**d_TEM_ (nm)**	**Pd loading wt.% (ICP)**	**% Pd^0^ (XPS)**	**% Pd^δ+^ (XPS)**
Pd/CB	3.3 ± 0.9	2.7	43	57
Pd/CB(t)	3.3 ± 0.8	-	81	19
Pd/Vulcan	2.6 ± 0.7	3.0	57	43
Pd/Vulcan(t)	3.5 ± 1.6	-	82	18
Pd/MWCNT	2.5 ± 0.9	2.4	60	40
Pd/MWCNT(t)	4.0 ± 1.9	-	78	22

**Figure 4:**
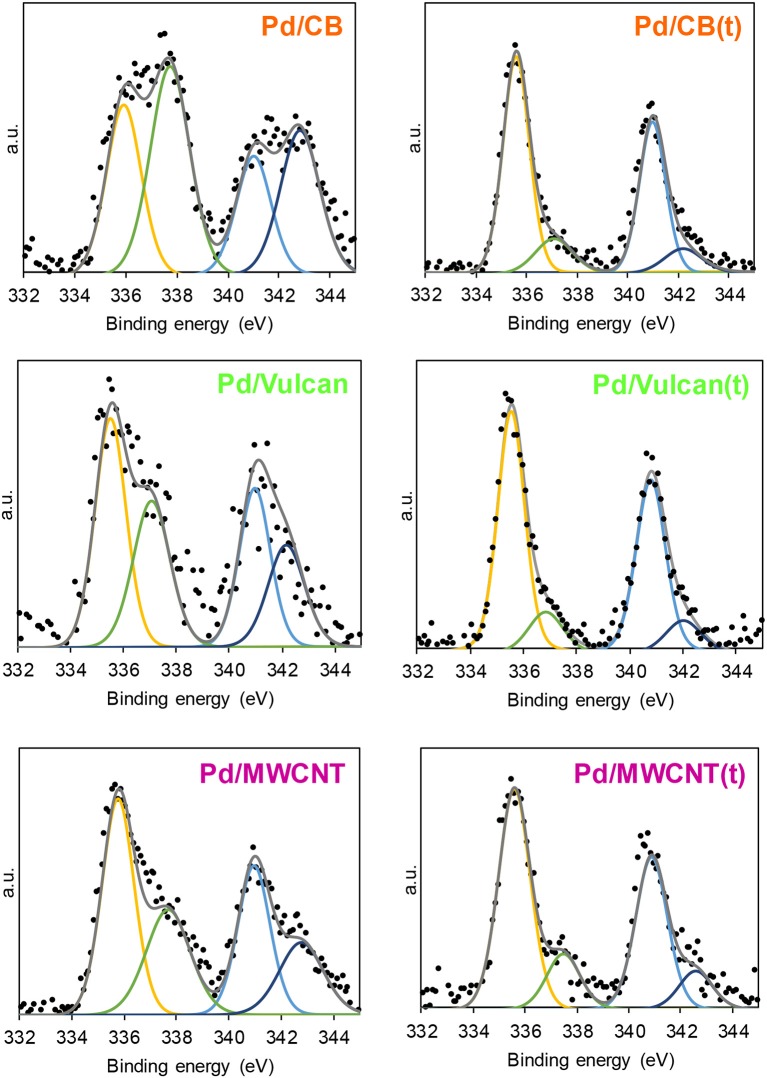
XPS spectra of Pd 3d.

XPS analysis was performed to investigate the electronic properties of the nanoparticles in the as-synthetized and PVP-free catalysts. A typical XPS Pd 3d spectrum shows two bands that correspond to the 3d_3/2_ (at higher binding energies) and 3d_5/2_ (at lower binding energies) transitions. Each band can be deconvoluted into two different contributions, associated with electronically different Pd species (Pd^0^ at lower binding energies and oxidized Pd (Pd^δ+^) at higher binding energies) (see Figure 4).

As observed in the results listed in Table 2, Pd in metallic and oxidized forms are present in all cases. The relative proportion of Pd^0^ increased considerably upon PVP removal, which confirms the electron withdrawal effect exerted by PVP molecules. Such an effect has also been observed in our previous studies and it was attributed to the interaction between Pd surface and PVP molecules via carbonyl group, which results in the presence of electron-deficient states or Pd^δ+^ (Miguel-García et al., 2010; García-Aguilar et al., 2016; Navlani-García et al., 2016a,c). Therefore, the increase of the relative content of Pd^0^ in the surface of the nanoparticles observed for the heat-treated catalysts is a consequence of the PVP removal from their surface.

### Catalytic Activity

**Figure 5:**
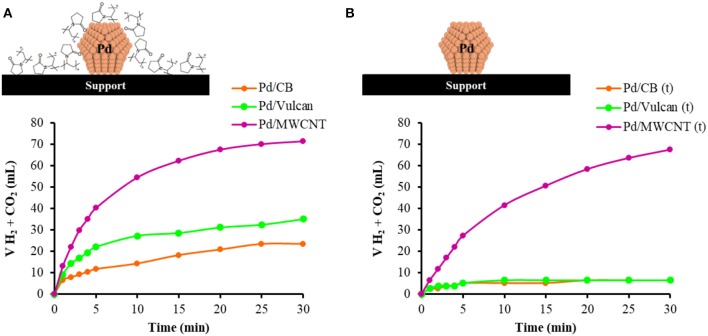
Gas evolution profiles achieved with **(A)** as-synthesized catalysts, and **(B)** PVP-free catalysts.

The catalytic ability toward the production of hydrogen from FA was assessed by monitoring the gas evolution profiles achieved with the as-synthesized and the heat-treated catalysts. Figure 5 includes the profiles obtained with the two sets of catalysts. Concerning the as-synthesized (PVP-containing) catalysts, no induction time was observed and the gas evolution proceeded smoothly with time until reaching a gas production of 23.4, 35.1, and 71.5 mL of gas after 30 min of reaction for Pd/CB, Pd/Vulcan, and Pd/MWCNT, respectively. The volume of H_2_ generated corresponds to initial TOF values of 229, 288, and 515 h^−1^, respectively. The TOF values with respect to the measured surface Pd are 839, 833, and 1430 h^−1^, respectively. The catalytic performance significantly decayed for the PVP-free catalysts, achieving a gas production after 30 min of the reaction of 67.6 mL for Pd/MWCNT(t), and 6.5 mL for both Pd/CB(t) and Pd/Vulcan(t). It must be noted that, even though the performance of the Pd/MWCNT(t) catalyst is the closest to the non-treated counterpart and very similar volume of gas is generated at the end of the catalytic test with both samples, the initial activity of Pd/MWCNT and Pd/MWCNT(t) is very different [TOF values of 515 and 257 h^−1^ for Pd/MWCNT and Pd/MWCNT(t), respectively], which evidences the superior performance of the PVP-containing catalyst.

The better behavior shown by PVP-containing catalysts might be related to several factors. On the one hand, the presence of PVP molecules might endow the surface of the catalysts with N-containing groups, which are beneficial to catalyze the dehydrogenation of formic acid (Martis et al., 2013; Bulushev et al., 2016a; Navlani-García et al., 2018b; Salinas-Torres et al., 2019). The basic N-groups can increase the local concentration of formic acid, thus increasing the reaction rate. It is expected that the most probable interaction between PVP molecules and the reactant is between the N-groups of PVP and the acidic proton of formic acid molecules. On the other hand, the presence of PVP would also increase the hydrophilicity of the materials, favoring, therefore, their dispersion in the reaction medium and enhancing the interface contact between the reactant molecules and the catalytic active sites. An additional positive effect of the PVP-containing catalysts might be the less accessible Pd atoms on the surface of the nanoparticles due to the interaction with PVP molecules. Such an interaction, which could be initially considered as negative because of the partial blocking of the possible active sites, would make more difficult the adsorption of reaction intermediates (i.e., CO_2_, H_2_O, and/or HCOO^−^), thus avoiding the repulsive adsorbate-adsorbate interaction and enhancing the catalytic performance (García-Aguilar et al., 2016).

Although recent studies have reported higher TOF values with some sophisticated catalytic systems and more tedious experimental protocols used for the syntheses of catalysts, the values achieved in the present study are competitive with those addressed for other catalysts [Pd/C synthesized with citric acid: 64 h^−1^ at 25°C (Wang et al., 2012); Pd/1.0Ti-g-C_3_N_4_: 77 h^−1^ at 30°C (Wu et al., 2017); Pd/H-BETA(0.5): 59.2 h^−1^ at 50°C (Navlani-García et al., 2015a); commercial Pd/C: 339 h^−1^ at 60°C (Hu et al., 2014); Ag_18_Pd_82_@ZIF-8: 580 h^−1^ at 80°C (Dai et al., 2015); Pd–Au/C: 27 h^−1^ at 92°C (Zhou et al., 2008), etc.].

To understand the tendencies displayed by the two sets of catalysts, several factors should be considered. The difference in the catalytic performance attained by a certain system is normally related to their features (i.e., size and electronic properties of the nanoparticles, porous texture of the catalysts, location of the nanoparticles, accessibility to reactant and interaction with the reaction products, etc.). It has been reported in other studies that the size of the nanoparticles has an important impact on the catalytic performance; however, different tendencies have been observed (Navlani-García et al., 2016b; Kim and Kim, 2019). In this case, better performances were observed for smaller nanoparticles (TOF of 229, 288, and 515 h^−1^ for nanoparticles of 3.3, 2.6, and 2.5 nm, respectively) for the as-synthesized catalysts. Nevertheless, such trend was not consistent with that displayed by PVP-free catalysts, since Pd/MWCNT(t), with the largest average nanoparticle size among investigated (4.0 nm), presented the best activity within PVP-free set of catalysts, suggesting that the size of the nanoparticles is not the main factor in governing the catalytic performance of the systems under investigation. Concerning the electronic properties of the nanoparticles, several investigations have reported on the positive effect of electron-rich Pd species in catalyzing the decomposition of FA, which has been particularly emphasized by using Pd-based alloyed nanoparticles (Mori et al., 2013; Navlani-García et al., 2016c; Wen et al., 2019). In the present study, the larger relative proportion of Pd(0) in Pd/MWCNT might be related to the better performance displayed by that catalyst as compared to Pd/Vulcan and Pd/CB. However, the catalytic activity decay seen for PVP-free catalysts with larger relative proportion of Pd(0) compared to PVP-containing counterpart might indicate that, under the experimental condition used in this study, this aspect is not the key factor in controlling the activity of the catalysts.

Apart from the properties of the nanoparticles, the nature and properties of the support is another factor to bear in mind while analyzing the performance of the resulting materials. The apparent surface area of the support is a well-known factor to be considered while aiming at synthesizing highly dispersed supported nanoparticles. However, the importance of such factor might be a bit blurred while using pre-synthesized colloidal nanoparticles instead of standard impregnation methods. Besides the apparent external surface area, the porous texture (in terms of pore size and pore size distribution) would be another point of interest. In this line, previous studies evidenced the importance of the presence of mesopores in attaining promising performance in the decomposition of FA (Navlani-García et al., 2018b). If porous texture was the only considered factor, Pd/CB catalyst should display a better performance by virtue of the larger apparent surface area and pore volume of CB as compared to the other supports. However, attending to the decrease of the S_BET_ and volume of pores observed in Pd/CB as compared to the bare CB support (S_BET_ of 604 and 126 m^2^/g, and V_DR_ of 0.38 and 0.05 cm^3^/g, and V_meso_ of 0.42 and 0.20 cm^3^/g, for CB and Pd/CB, respectively) it could be said that part of the nanoparticles are partially blocking the pores of CB, which is consistent with the changes observed in the pore size distribution of CB upon Pd loading. It is important to note that while such partial blockage or filling of the porosity is observed in the three catalysts, the decrease in the apparent external surface area upon Pd loading is much more significant for CB (decrease of S_BET_ after Pd loading of 79, 69, and 42% for Pd/CB, Pd/Vulcan, and Pd/MWCNT, respectively).

According to the above results, Pd/MWCNT is the most promising catalyst among investigated and under the experimental conditions used in this study. The beneficial effect of using MWCNTs as support might be mainly related to their 1D structure and high availability of the surface area, which has previously been detected to be a key aspect in achieving good catalytic activities toward the dehydrogenation of formic acid (Masuda et al., 2018; Navlani-García et al., 2018b). The lower contribution of micropores in Pd/MWCNT compared to the other catalysts might result in less diffusion problems and the subsequent better catalytic performance. It should be mentioned that while the use of MWCNTs for the preparation of metal-supported catalysts for the electrooxidation of formic acid has been fruitfully investigated (Zhang et al., 2009; Chakraborty and Raj, 2010; Chen et al., 2010; Morales-Acosta et al., 2010; Winjobi et al., 2010), their use for the thermal decomposition of formic acid is much less explored than in the case of other carbon materials. The results herein enclosed indicate that MWCNTs are a suitable candidate as support of Pd nanoparticles for the preparation of efficient catalysts to produce hydrogen from the thermal decomposition of formic acid. It was also observed that the incorporation of PVP as a capping agent of the supported metal nanoparticles could be an easy strategy for the incorporation of N-groups in the catalysts while avoiding more sophisticated and tedious experimental procedures. Unfortunately, the catalysts studied in this work lack of good stability under reaction conditions and, therefore, further improvements are needed to achieve highly-performance and stable catalysts for the dehydrogenation of formic acid.

## Conclusions

Catalysts based on PVP-capped Pd nanoparticles supported on various carbon materials have been synthesized and assessed in the decomposition of formic acid in the liquid phase. Among investigated, MWCNT-supported catalysts displayed the best performance, which might be related to their 1D structure and highly available external surface area. In addition, PVP-Pd-based catalysts showed better activities compared to the counterpart PVP-free samples, which could be ascribed to the increase of hydrophilicity of the materials and interaction with formic acid and the favored dispersion of the catalysts in the reaction medium that ultimately enhances the interface contact between the reactant molecules and the catalytic active sites. It could be envisaged that the selection of different capping agents and catalytic supports with tailored porous structure would give rise to promising catalytic systems for the present application. Although much effort is still needed to achieve the optimum catalysts and fully understand all the pivotal factors to be considered while optimizing the catalysts for the dehydrogenation of formic acid, it could be expected that the present study will pave the way for the design of efficient functionalized carbon-material-based catalytic systems prepared by simple experimental protocols.

## Data Availability Statement

The datasets generated for this study are available on request to the corresponding author.

## Author Contributions

AO-M synthesized and characterized the catalysts and helped with the analysis of the results. MN-G designed and performed the experiments corresponding to catalytic tests, analyzed the results and wrote the manuscript. EM and DC-A contributed to the conception of the study and helped with the discussion of the results as well as with the revision of the manuscript. All authors approved the manuscript for publication.

## Conflict of Interest

The authors declare that the research was conducted in the absence of any commercial or financial relationships that could be construed as a potential conflict of interest.
