# Supplementary material for: MWCNT-Supported PVP-Capped Pd Nanoparticles as Efficient Catalysts for the Dehydrogenation of Formic Acid
Source: Front Chem. 2020 Apr 28;8:359. doi: 10.3389/fchem.2020.00359 (PMC7199183; doi:10.3389/fchem.2020.00359)
Supplement: Supplementary file 1 [file Table_1.DOCX]

*Supporting Information*

MWCNT-supported PVP-capped Pd nanoparticles as efficient catalysts for the dehydrogenation of formic acid

Alejandro Ortega-Murcia^1^, Miriam Navlani-García^2,*^, Emilia Morallón^1^, Diego Cazorla-Amorós^2^

^1^ Physical Chemistry Department, Institute of Materials Science (IUMA), University of Alicante (UA), Ap. 99, Alicante, 03080, Spain

^2^ Inorganic Chemistry Department, Institute of Materials Science (IUMA), University of Alicante (UA), Ap. 99, Alicante, 03080, Spain

*** Correspondence:**Corresponding Author
miriam.navlani@ua.es

**Figure S1.** Additional TEM micrographs to show more information about the nanoparticle distribution.
